# Ultrafast myoglobin structural dynamics observed with an X-ray free-electron laser

**DOI:** 10.1038/ncomms7772

**Published:** 2015-04-02

**Authors:** Matteo Levantino, Giorgio Schirò, Henrik Till Lemke, Grazia Cottone, James Michael Glownia, Diling Zhu, Mathieu Chollet, Hyotcherl Ihee, Antonio Cupane, Marco Cammarata

**Affiliations:** 1 Department of Physics and Chemistry, University of Palermo, Palermo 90128, Italy; 2 CNRS, Université Grenoble Alpes, CEA—Institut de Biologie Structurale, Grenoble 38044, France; 3 LCLS, SLAC National Accelerator Laboratory, Menlo Park, California 94025, USA; 4 Center for Nanomaterials and Chemical Reactions, Institute for Basic Science (IBS), Daejeon 305-701, Republic of Korea; 5 Department of Chemistry, KAIST, Daejeon 305-701, Republic of Korea; 6 Department of Physics, UMR UR1-CNRS 6251, University of Rennes 1, Rennes 35042, France

## Abstract

Light absorption can trigger biologically relevant protein conformational changes. The light-induced structural rearrangement at the level of a photoexcited chromophore is known to occur in the femtosecond timescale and is expected to propagate through the protein as a quake-like intramolecular motion. Here we report direct experimental evidence of such ‘proteinquake’ observed in myoglobin through femtosecond X-ray solution scattering measurements performed at the Linac Coherent Light Source X-ray free-electron laser. An ultrafast increase of myoglobin radius of gyration occurs within 1 picosecond and is followed by a delayed protein expansion. As the system approaches equilibrium it undergoes damped oscillations with a ~3.6-picosecond time period. Our results unambiguously show how initially localized chemical changes can propagate at the level of the global protein conformation in the picosecond timescale.

## Abstract (web summary)

Localized chemical events such as the breakage of a bond between a protein and a ligand may trigger a global protein conformational change. Here, the authors use an X-ray free-electron laser to track the motion of myoglobin in response to photoinduced ligand release, and observe a picosecond proteinquake.

## Graphical abstract

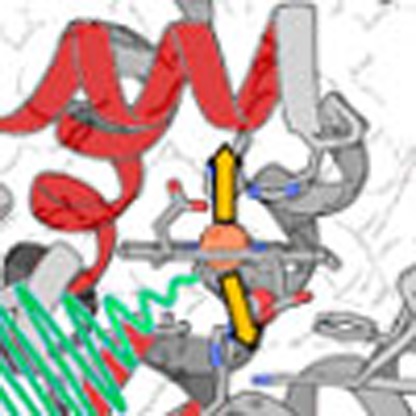

Chemical reactions often involve the motion of both electrons and nuclei of the participating molecules. In the case of proteins, the molecular machines of living organisms, the study of the dynamics of molecular motions is particularly fascinating since localized ultrafast chemical events, such as bond breaking, may trigger biologically relevant concerted motions of thousands of atoms usually referred to as protein conformational changes^1^. Many studies have shown that proteins are dynamic objects with a hierarchy of various intramolecular motions spanning a wide range of time and length scales^1^,^2^. In this regard, photosensitive proteins, which can be excited with ultrashort light pulses, serve as excellent model systems since they allow studying dynamics in a wide time window extending from a few tens of femtoseconds to seconds or more^3^. Many of such studies have used myoglobin (Mb), a relatively small protein (~18 kDa) that has been named the hydrogen atom of biology^4^ and has played a central role for our understanding of protein dynamics^5^. Mb is a monomeric protein consisting of 153 amino acids folded into eight α-helices connected by short loops. Small ligands such as molecular oxygen (O_2_) and carbon monoxide (CO) are able to bind reversibly to Mb and the bond between the ligand and the haem molecule contained in the active site of the protein can be photolyzed with high efficiency within 50 fs^6^. The events following photolysis have been extensively studied with a variety of techniques such as time-resolved optical absorption^7^,^8^,^9^,^10^, circular dichroism^11^, resonance Raman^12^,^13^,^14^, photoacoustic calorimetry^15^ and transient grating spectroscopy^16^,^17^. The results of these investigations indicate that an initial ultrafast rearrangement of the haem molecule, which ensues a so-called doming of the haem structure^18^ and an out-of-haem-plane motion of the central iron ion^19^, triggers a series of structural changes that extend from the amino acids close to the haem through the entire polypeptide chain and solvent hydration layer^20^. The initial ultrafast protein response to the breaking of the bond between the ligand and the protein has been described as a quake-like motion of Mb, since the propagation of the strain released upon photoexcitation through the protein is similar to the propagation of acoustic waves during an earthquake^21^. Such ‘proteinquake’ model has been used to describe the structural dynamics of other haem^22^ and non-haem proteins^23^,^24^,^25^. The analysis of the elastic response of the protein to the active site rearrangement is complicated by the simultaneous dissipation of the excess energy that is deposited by the photolysis pulse on the haem chromophore. Transient resonance Raman experiments^19^,^26^ and molecular dynamics simulations^27^ have demonstrated that most of haem cooling occurs within a few ps mostly through direct haem-solvent energy transfer. Dissipation of residual excess kinetic energy occurs through the polypeptide chain at a longer timescale (few tens of picoseconds) as demonstrated by transient grating spectroscopy^28^. Moreover, analogous experiments performed on deoxyMb^16^ have suggested the non-thermal origin of the quake-like response observed after photolysis of carbonmonoxy myoglobin (MbCO). A first experimental evidence of a proteinquake based on the use of a direct structural probe has been recently reported by Arnlund *et al.*^29^ in the case of multiphoton excitation of a bacterial photoreaction centre. These authors have used time-resolved X-ray solution scattering, an experimental technique able to track the structural dynamics of proteins in solution^30^, to show that the backbone carbon atoms of the protein helices increase their distance from the interior of the photoreaction centre on a picosecond timescale. However, under the extensive protein multiphoton absorption conditions employed, protein motions occurred in the presence of a very large excess energy deposited in the system, and the observed protein response may not reflect physiologically relevant protein functioning conditions. In the case of Mb, the experimental investigations performed so far were based either on spectroscopic measurements or did not have sufficient time resolution to measure the timescale of the proteinquake. Indeed, time-resolved X-ray solution scattering recently performed on Mb have demonstrated^31^, ^32^, ^33^ that, although subtle protein structural changes have been observed in the nanoseconds-to-microseconds timescale, the most significant global structural rearrangements, corresponding to a relative motion of helices^33^ (as revealed by changes in the wide-angle X-ray scattering (WAXS) signal) and an estimated 22 Å^3^ volume change^31^ (as probed with small-angle X-ray scattering (SAXS)), occur within the time resolution of 100 ps available in those experiments, and in agreement with previous transient grating spectroscopy results^17^.

Here, we use the femtosecond X-ray pulses produced by the Linear Coherent Light Source (LCLS) X-ray free-electron laser (X-FEL) to visualize the structural response of Mb after photolysis. We show how the perturbation at the active site level propagates to the global protein structure with a timescale set by the acoustic speed of sound, thus confirming the proteinquake hypothesis^21^. The time evolution of both the radius of gyration and the volume of Mb reveals an oscillatory collective motion of the protein atoms that is damped in few ps, thus highlighting the relevance of underdamped low-frequency vibrations in proteins. Our data illustrate how ultrafast studies are potentially able to capture the intrinsic ballistic-like nature of protein motion that is generally hidden in ensemble measurements at longer timescales.

## Results

### Time-resolved X-ray scattering difference patterns

**Figure 1:**
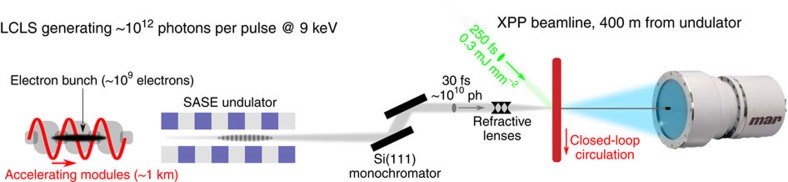
Experimental set-up. The sample (a 2.35-mM MbCO solution) was circulated in a closed loop through a fused-silica capillary (0.3 mm diameter) positioned at the overlap between the X-ray pulses (9 keV, 30 fs) produced by the LCLS (~10^10^ photons per pulse at 9 keV are transmitted by a silicon monochromator) and the photolysis pulses (538 nm, 250 fs) produced by the optical laser system (~0.3 mJ mm^−2^). By monitoring the pattern of the X-rays scattered by the sample at different time delays between the optical and X-ray pulses, it was possible to track the structural changes occurring in the sample after photolysis.

**Figure 2:**
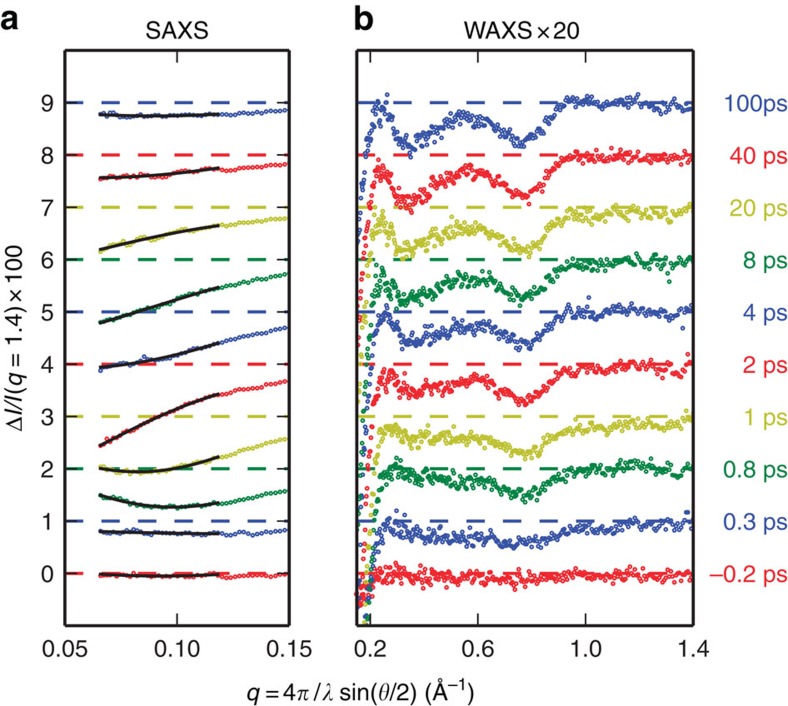
Photolysis-induced time-resolved X-ray scattering difference patterns for MbCO in solution. A significant change in both intensity and shape is evident in the SAXS region (**a**); continuous black lines are fittings in terms of Guinier analysis (see Methods). Data in the WAXS region (**b**, magnified by a factor 20) show a difference signal that develops within 1 ps and then changes in shape, while increasing in amplitude, with a timescale of the order of a few picoseconds. Data have been vertically offset for clarity.

To decipher the exact timescale of the ultrafast structural dynamics in Mb, we have performed femtosecond time-resolved X-ray solution scattering experiments on Mb at the LCLS X-FEL (Fig. 1, see Methods). Laser-induced time-resolved difference scattering curves obtained by photoexciting a solution of horse MbCO with 250 fs pulses at 538 nm are shown in Fig. 2.

The probe X-ray pulses (~30 fs long) hit the sample at several time delays after photolysis, spanning a time range up to 100 ps with a time resolution of ~500 fs (see Methods). Changes in the X-ray scattering signal have been simultaneously monitored both in the SAXS and WAXS regions. The overall shape of the difference signals is similar to what has been observed in the past for Mb with a much lower (100 ps) time resolution^30^,^31^,^32^,^33^, with the 100 ps difference pattern measured in the present experiment (see Fig. 2) being essentially identical to what has been reported in the literature^31^,^32^. The signal changes in shape and grows in intensity mostly within 10 ps, and a clear difference signal is observed both in the SAXS and WAXS regions already at 0.8 ps from photolysis.

### Analysis of the X-ray scattering signal time evolution

**Figure 3:**
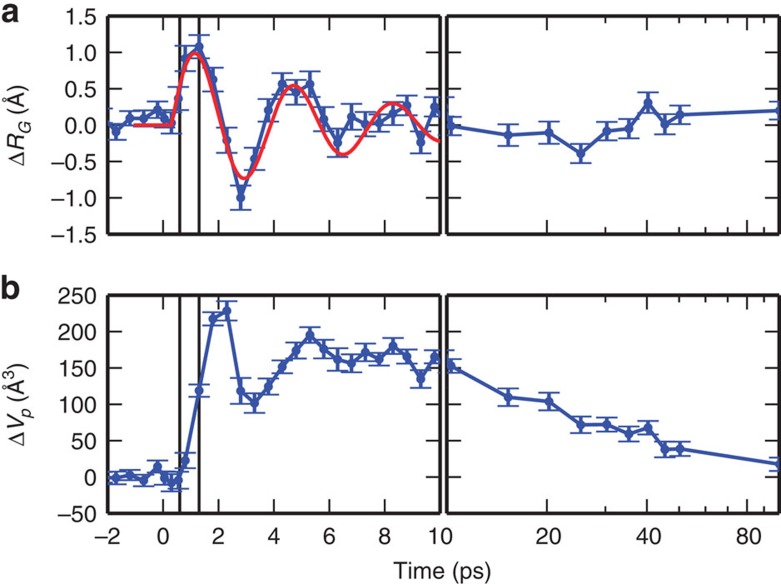
Time-dependent changes of protein structural parameters. The analysis of time-resolved SAXS data (blue symbols) reveals that *R*_g_ increases by ~1 Å within 1 ps and exhibits damped oscillations around its equilibrium value (**a**; the red curve represents a damped oscillation with a 3.6 ps time period and 6 ps decay time). The increase in the protein volume (**b**) is delayed with respect to the *R*_g_ increase: *V*_p_ increases by 220 Å^3^ within 2 ps. The observation of a delayed volume expansion implies that an initial ultrafast redistribution of protein mass from the active site towards the outside solvent (as measured by the *R*_g_ increase) occurs without significant volume change within 1 ps. After few tens of ps, *V*_p_ relaxes to a value slightly higher with respect to that before photoexcitation (Δ*V*_p_~20 Å^3^ at 100 ps). Error bars are from the fitting procedure used to obtain Δ*R*_g_ and Δ*V*_p_, and correspond to one s.d. The black vertical lines are guides to the eye and they are spaced by 0.7 ps.

The radius of gyration (*R*_g_) and volume (*V*_p_) of proteins can be directly obtained from the SAXS signal^34^ (solid black lines in Fig. 2, see Methods). The ultrafast increase of *R*_g_ at nearly constant *V*_p_ (Fig. 3, top panel) and the subsequent increase in *V*_p_ (Fig. 3, bottom panel), which is delayed by ~1 ps, clearly shows that a change in the mass distribution of the protein precedes its expansion (see Supplementary Fig. 1 for a comparison of analyses based on different data sets). Considering the overall size of Mb (*R*_g_~17 Å), this implies that the strain released upon photolysis roughly propagates at ~20 Å ps^−1^, in agreement with the expected speed of sound in proteins^35^. Analogous timescales are observed in the WAXS region, where a clear ultrafast difference signal (negative peak at ~0.75 Å^−1^) appears within 1 ps from photoexcitation and it further changes in shape with a negative peak at ~0.3 Å^−1^ developing in few ps. The simultaneous evolution of both SAXS and WAXS signals shows that the motion of secondary structure elements is responsible for the *R*_g_ and *V*_p_ changes. In light of our data, it is clear that the global conformational change reflected in the WAXS difference signal, already attributed to the relative motion of protein helices^30^,^31^,^32^,^33^ and in analogy to what has been found with time-resolved WAXS measurements on other photosensitive proteins^21^,^36^,^37^, is essentially completed in 10 ps. As already demonstrated in previous studies^7^,^8^,^9^,^10^,^31^,^32^,^38^, further more subtle structural changes, which complete the conformational relaxation towards the unbound Mb equilibrium state, occur at longer timescales (extending from a few nanoseconds to a few microseconds).

## Discussion

The results here presented demonstrate that Mb undergoes significant global structural changes in the ps timescale after photolysis of the bond between the protein and the CO ligand. At difference with the recent report by Arnlund *et al.*^29^, by monitoring simultaneously both the SAXS and WAXS region, we have been able to extract directly the time evolution of relevant structural parameters. Our data clearly show that, even in biophysically relevant photoexcitation conditions (we estimate that, in our experimental conditions, ~1.6 photons are absorbed per chromophore within the duration of a photolysis pulse), an ultrafast proteinquake is observed.

**Figure 4:**
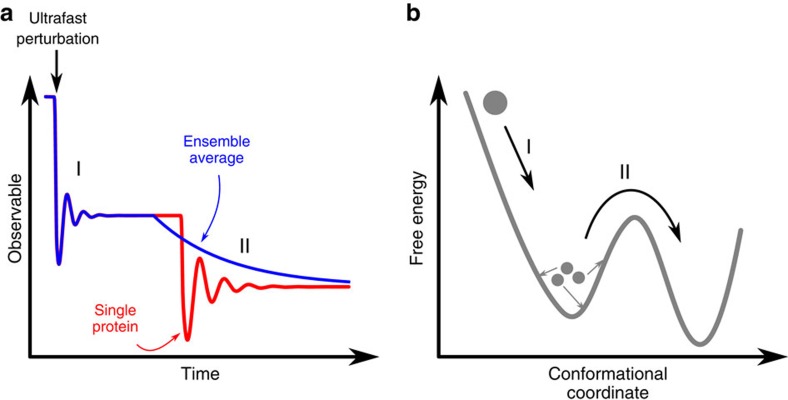
Effect of ensemble average on an experimental observable after an ultrafast perturbation. We postulate that elementary motions at the level of a single protein have a quake-like ballistic nature. (**a**) Time evolution of an observable in an idealized single-molecule experiment (red curve). Experiments on large protein ensembles (blue curve) are able to detect such quake-like transitions only at very short times (step I), when the initial synchronization established by the photoexcitation pulse is still preserved. At longer times (step II), the observed time evolution is dominated by thermally activated transitions from one protein state to the other, which result in the ‘exponential-like’ kinetics typically observed in most time-resolved experiments. (**b**) Simplified free-energy diagram illustrating the difference between a downhill transition along the energy landscape (step I) and a thermally activated process; in the latter case, different protein molecules will undergo the same structural transition but with a statistically distributed onset.

Such an ultrafast, ballistic-like response of the protein might seem to contrast with the exponential-like time dependencies that typically characterize protein relaxations at longer timescales. Indeed, kinetic experiments on protein ensembles typically probe conformational relaxations that are dominated by thermally activated steps and do not reflect the actual relaxation of individual proteins, but rather the average timescale of energy barriers crossing. In this respect, ultrafast experiments using extremely short photoexcitation pulses have the advantage of unveiling the intrinsic nature of protein elementary motions before thermally activated processes start to play a role (Fig. 4). Similar evidence of such elementary stepwise protein motions have been recently obtained with ultrafast infrared spectroscopy^39^ and single-molecule fluorescence resonance energy transfer experiments^40^. We speculate that individual ballistic-like motions (e.g., one for each bond formation/breaking), rather than gradual structural transitions from one protein state to the other, could be a general feature of protein functional motions, although more experiments will be needed to confirm such a hypothesis.

**Figure 5:**
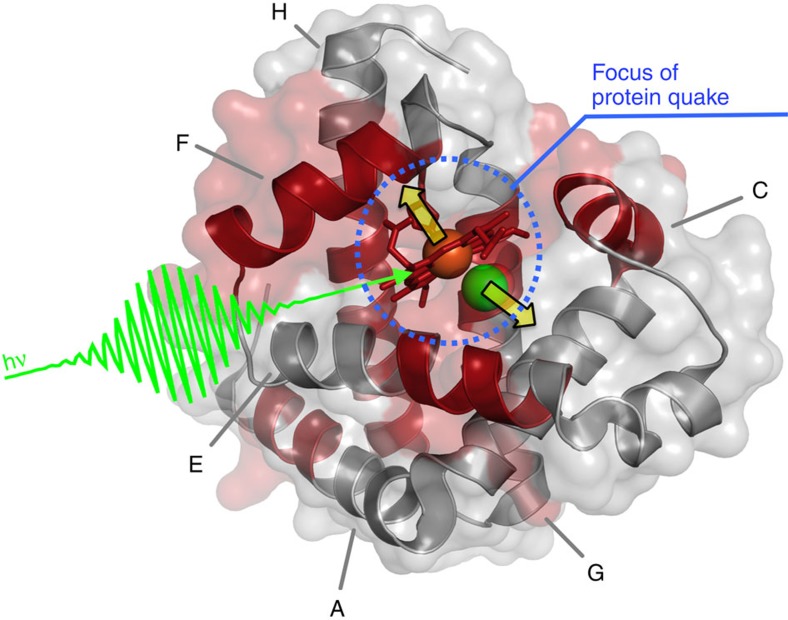
Myoglobin proteinquake. Cartoon representation of the Mb three-dimensional structure; the segments of the polypeptide chain contributing to the collective mode having a ~4 ps period (which is activated by ligand release) are highlighted in red (see Methods for details). This vibrational mode involves the haem, the helix to which the haem is covalently bound (F), and parts of the A, C, E, G and H helices. Note that the regions involved participate to define the haem pocket and extend to the protein surface.

Another important result of our study is that the Mb structure oscillates with a period of ~3.6 ps as evidenced by the time dependence of *R*_g_ and *V*_p_. Interestingly, a collective vibrational mode of Mb with a comparable time period (~4 ps) has been suggested to be strongly coupled to the conformation of haem and of the proximal histidine and is activated by the strain release induced by photolysis^41^. Figure 5 illustrates how the collective mode involves large protein regions from the active site to the protein surface. Such coupling with the mode at ~4 ps was already suggested on the basis of transient grating investigations^17^, although oscillations of the Mb structure could not be detected in those experiments.

The observation of oscillations in Mb structural parameters (*R*_g_ and *V*_p_) demonstrates that the elastic response of Mb is not dominated, at least in the ultrafast timescale, by damping mechanisms. Although evidences of underdamped protein collective motions have been recently obtained with THz spectroscopy^42^, this is a surprising result since theoretical arguments would suggest that low-frequency (<50 cm^−1^) protein oscillations are overdamped in a liquid environment. Indeed, a simple model approximating Mb as a homogeneous elastic sphere oscillating in water predicts a damping time of ~1.5 ps for an oscillation period close to that observed in our experiment (3.6 ps, see Methods). More sophisticated computational analysis based on atomic resolution protein models predict low-frequency protein collective motions to be overdamped^43^. At difference, our data show that Mb can also undergo underdamped collective vibrations at ~10 cm^−1^ after the bond between the protein and the ligand is broken.

The results here presented demonstrate that the combination of sub-picosecond time resolution available at X-FEL sources and the direct structural sensitivity of X-ray solution scattering techniques can be advantageously used to investigate the intrinsic nature of protein elementary motions.

From the point of view of biology, the present study may be of general relevance for understanding the structural basis of biological reactions that involve ultrafast chemical processes, as it is the case of electron transfer in photosynthesis. It is likely that the initial primary charge separation events, which are known to be photoinduced and occur in the picosecond timescale, are modulated by global protein motions analogous to those reported for Mb in the present paper.

## Methods

### Sample preparation and data acquisition

The experiment was performed at the X-ray Pump and probe (XPP) endstation of the LCLS X-FEL (SLAC National Accelerator Laboratory). The sample was a solution of horse MbCO in 0.1 M phosphate buffer at pH 7.4 with a protein concentration of 2.35 mM (~40 mg ml^−1^) and a threefold molar excess of sodium dithionite. The vial containing the 20 ml of CO-saturated Mb solution was connected to a liquid flowing system driven by an HPLC pump able to circulate the solution (3 ml min^−1^ flow speed) in a closed loop through a 0.3 mm fused-silica capillary (10 μm wall thickness) positioned at the intersection of the optical and X-ray laser beams. Photolysis of the bond between Mb and CO was achieved with circularly polarized pulses (~250 fs full-width at half-maximum; 25 μJ per pulse; 538 nm) focused to a 300 μm full-width at half-maximum circular spot (energy density ~0.3 mJ mm^−2^ at the sample), in nearly collinear (~1°) geometry. The absorbance of the sample at the pump wavelength was ~1.0 optical density. Given the size, energy of the photolysis beam, the concentration and molar extinction coefficient of the haem, ~1.6 photons are expected to be absorbed by each haem molecule at each photolysis pulse. A monochromatic X-ray beam at 9 keV was produced by a Si(111) double-crystal monochromator and focused to 100 μm by beryllium refractive lenses. Time-resolved X-ray scattering images were acquired with a two-dimensional charge-coupled device detector (Rayonix SX165, 2,048 × 2,048 pixels) at 16.8 cm from the sample. Each image is the result of the accumulation of 360 X-ray shots. Investigated time delays ranged between −3 and 100 ps (with negative time delays corresponding to a probe pulse arriving before the photolysis one). In particular, we measured every 0.5 ps between −3 and 10 ps; every 5 ps between 10 and 50 ps; every 50 ps at longer time delays. Reference images at a time delay of −100 ps were also recorded every 7 other images. The scattering pattern at each time delay is the result of an average over 50 repetitions. The repetition rate of the experiment was 120 Hz and the estimated time resolution was ~500 fs.

### Experimental time resolution

The relative timing between X-ray and visible pulses has been monitored using the timing tool developed^44^ at the XPP endstation of the LCLS X-FEL^45^, which exploits the ultrafast free-carrier generation induced by X-rays in a Si_3_N_4_ membrane to encode the relative arrival time of X-ray and visible pulses. The timing tool data show that over the ~6 h of data collection the X-ray/laser timing has been stable to about ~150 fs and that the jitter was about 200 fs. The time resolution of the experiment is also affected by the group velocity mismatch between the X-ray and optical pulses propagating inside the 300 μm thick protein solution. By taking into account both effects and the duration of photolysis pulses (~250 fs), we estimate an overall time resolution of ~500 fs.

### Calculation of scattering difference patterns

Two-dimensional scattering images recorded with a Rayonix SX165 detector were azimuthally averaged to give one-dimensional scattering patterns. The scattering angle *θ* was converted to momentum transfer *q* using the formula *q*=4*π*/*λ*sin(*θ*/2), where *λ*=1.377 Å is the X-rays wavelength. Since the changes induced in the scattering patterns by optical photoexcitation account for less than a percent of the absolute signal, data were normalized^30^ at 1.4±0.1 Å^−1^ before calculating difference patterns with respect to the pattern measured at −100 ps. For a given time delay, each scattering difference is compared with the average difference and a simple reduced 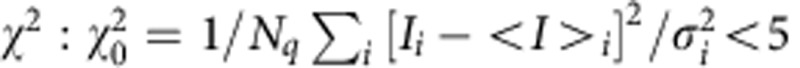 criterion is used to discard the differences that are too far away from the average one. In the formula above, *I*_*i*_ refers to the scattered intensity for a given time delay and q-bin, *I*_*i*_ is the scattered intensity averaged over all repetitions, and *σ*_*i*_ is the error bar on the experimentally measured intensity. The differences that satisfy the above criterion are then used to produce the averages difference patterns shown in the paper. This procedure usually rejects ~15–25% of the differences.

### Analysis of SAXS data

The scattered intensity of a dilute protein solution in the small-angle region can be approximated by the following expression^46^:


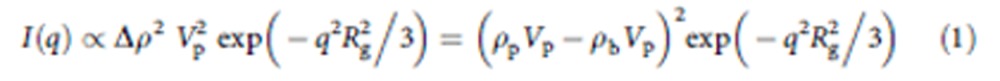


where Δ*ρ*=*ρ*_p_*—ρ*_b_ is the electron density difference between the protein and surrounding buffer solution, *V*_p_ is the protein volume, *q* is the magnitude of the scattering vector and *R*_g_ is the protein radius of gyration. A conformational change of the protein may affect *V*_p_ and *R*_g_, but not the total number of electrons it contains (*ρ*_p_*V*_p_) that has thus been kept constant (~9,900 electrons) in the fits of SAXS signals. Since the experimental data are SAXS difference patterns, they have been fitted using the following expression:


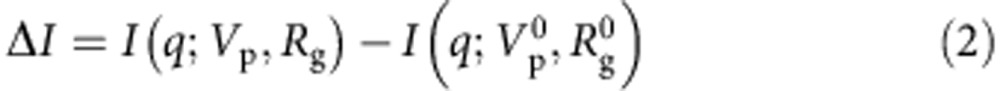


with 
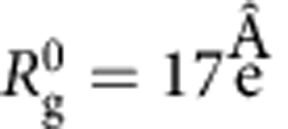
, 
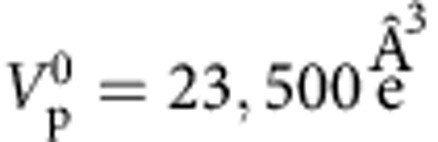
 and *ρ*_b_=0.34 electron Å^−3^.

### Representation of Mb activated vibrational mode

To obtain a graphical representation of the residues most affected by the Mb collective mode having a ~4 ps period, we have followed the reasoning by Seno and Go^41^. Starting from their normal mode analysis, the authors have evaluated the magnitude of the ligand-induced conformational change by calculating, for each residue (including the haem molecule), the mass-weighted square atomic displacement over all the atoms belonging to the residue. In particular, we used the data reported in figure 8 of ref. 41 to calculate the involvement of each residue of the Mb sequence in the ~4 ps collective normal mode. The residues that have the highest values (>10% of the maximum) of the mass-weighted square displacements are depicted in red in Fig. 5.

### Elastic sphere model

To estimate the frequency and damping time of Mb vibrational modes, we have used a model that approximates the protein as an homogeneous elastic sphere oscillating in water. Under this approximation the frequencies and damping times can be calculated following the procedure described by Talati and Jha^47^. In particular, the (complex) normalized frequencies (*s*) have to satisfy the following equation:


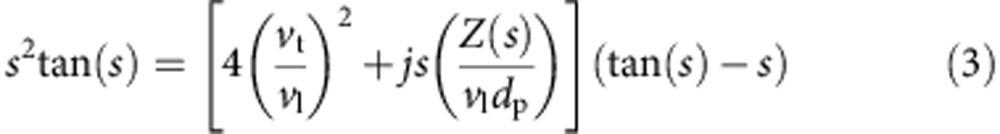


where *Z*(*s*) is the acoustic impedance, *j* the imaginary unit, *v*_l_ and *v*_t_ are the longitudinal and transverse speed of sound, respectively, in the protein and *d*_P_ is the protein mass density. The (complex) normalized frequency *s*=*ωR/v*_l_, where *ω* is the complex angular frequency and *R* is the spherical protein radius. The acoustic impedance *Z*(*s*) is equal to:


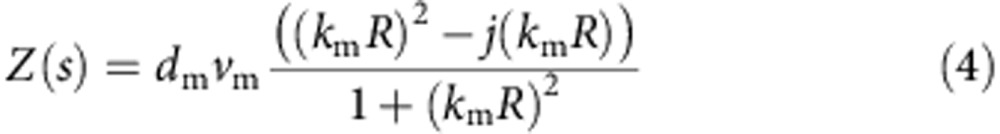


where *d*_m_ and *v*_m_ are the density and sound speed of the medium, respectively, surrounding the sphere and *k*_m_=*ω/v*_m_. Finally, the damping time *τ*_D_ and the complex angular frequency *ω* are related by the following expression: *τ*_D_=−Im(*ω*).

The relevant physical parameters used to perform the calculations are reported in Supplementary Table 1. The solution corresponding to the first three lowest-frequency vibrational modes are characterized by oscillation periods of 2.7, 1.15 and 0.75 ps, while the corresponding damping times are 1.3, 1.7 and 1.8 ps.

## Author contributions

M.Ca. conceived the project, H.T.L. and M.Ca. led the experiment preparation. M.L. G.S., H.T.L., J.M.G., D.Z., M.Ch. and M.Ca. performed the experiment. M.L., G.S. and M.Ca. analysed the data and wrote the manuscript with input from all authors.

## Additional information

**How to cite this article:** Levantino, M. *et al.* Ultrafast myoglobin structural dynamics observed with an X-ray free-electron laser. *Nat. Commun.* 6:6772 doi: 10.1038/ncomms7772 (2015).

## Supplementary Material

Supplementary Information Supplementary Figure 1 and Supplementary Table 1
