# Supplementary material for: Ultrafast myoglobin structural dynamics observed with an X-ray free-electron laser
Source: Nat Commun. 2015 Apr 2;6:6772. doi: 10.1038/ncomms7772 (PMC4396393; doi:10.1038/ncomms7772)
Supplement: Supplementary Information — Supplementary Figure 1 and Supplementary Table 1 [file ncomms7772-s1.pdf]

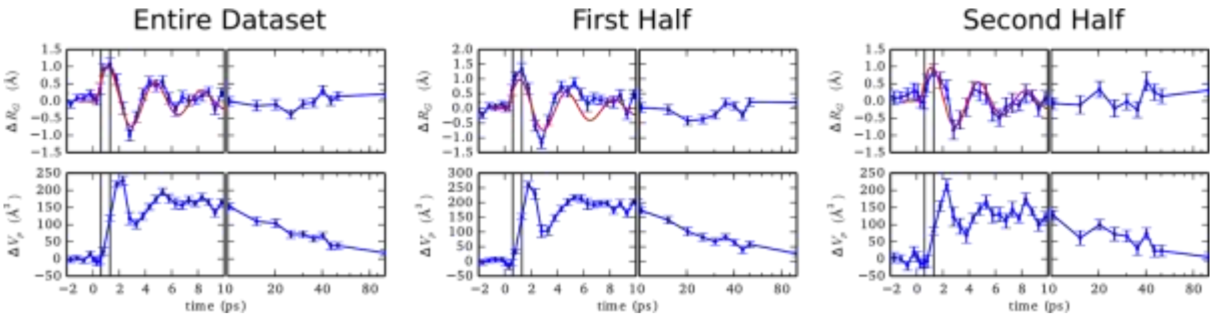

Supplementary Figure 1. Stability of the structural parameters retrieved from the analysis of the SAXS data. The changes in the protein radius of gyration (ΔR_g) and volume (ΔV_p) obtained with three different data reduction and analyses runs using the entire dataset (left panel, identical to Figure 3 of the main text), the first half dataset (middle panel) and the second half dataset (right panel) are compared. Despite minor discrepancies in the tail of the signal (at times longer than 6 ps) the above plots show that the main trend in the time evolution of both ΔR_g and ΔV_p is observed independently of the particular dataset chosen.

Supplementary Table 1. Physical parameters used for the elastic sphere model calculations.

Parameter	Value
R	22 Å
v_l	2000 m s^{-1}
v_t	1000 m s^{-1}
d_p	1370 kg m^{-3}
v_m	1483 m s^{-1}
d_m	1000 kg m^{-3}
